# Ethnomedicinal plants used to treat human ailments in the prehistoric place of Harla and Dengego valleys, eastern Ethiopia

**DOI:** 10.1186/1746-4269-10-18

**Published:** 2014-02-05

**Authors:** Anteneh Belayneh, Negussie F Bussa

**Affiliations:** 1 Department of Biology, College of Natural and Computational Sciences, Haramaya University, P.O. Box 282, Haramaya, Ethiopia; 2 College of Health and Medical Sciences, Haramaya University, P.O. Box 203, Haramaya, Ethiopia

**Keywords:** Dengego valley, Eastern Ethiopia, Ethnomedicinal knowledge, Harla, Traditional medicinal plants

## Abstract

**Background:**

Traditional medicines remained as the most affordable and easily accessible source of treatment in the primary health care system among diverse communities in Ethiopia. The Oromo community living in the prehistoric Harla and Dengego valleys has long history of ethnomedicinal know-how and practice against human and livestock ailments. However, this rich ethnomedicinal knowledge had been remained unexplored hitherto. This study focus on the comprehensive ethnomedicinal investigation in an attempt to safeguard the deteriorating ethnomedicinal knowledge that can be used as a steppingstone for phytochemical and pharmacological analysis.

**Methods:**

Fifty five (44 male and 11 female) systematically selected informants including ten traditional herbalists (key informants) were participated in the study. Semi-structured interviews, discussions and guided field walk constituted the data collection methods. Factor of informant consensus (*Fic*), frequency of citation (F%), and binomial test were employed in data analysis. Medicinal plant specimens were collected, identified and kept at Herbarium of Haramaya University (HHU).

**Results:**

A total of 83 traditional medicinal plant species against human ailments in 70 genera and 40 Families were recorded. Twelve medicinal plants were marketable in open market places of the nearby towns. Formulations recorded added to 140 remedies for 81 human ailments. Concoction accounts 50.7% of the total preparations followed by fluids extraction (10.7%) and infusion (6.4%). Fifteen different plant parts were used for remedies preparation wherein leaves accounted 46.4%, stem 9.2%, fruits and roots each 7.8%. Most of the remedies (90.7%) were prepared from single plant species like, aphrodisiac fresh rhizome of *Kleinia abyssinica* (A. Rich.) A. Berger chewed and swallowed few hours before sexual performance for a man having problem of erectile dysfunction. The *Fic* value ranges between 1.0 (gastritis and heartburn/pyrosis) and 0.77 (swollen body part). *Aloe harlana* Reynolds was reported to be used for the highest number of ailments treating swollen body part locally called GOFLA, colon cleaner, snake bite, liver swelling, spleen swelling/splenomegaly, fungal infections and inflammation of skin.

**Conclusion:**

Such documentation of comprehensive ethnomedicinal knowledge is very valuable and needs to be scaled-up so that it could be followed up with phytochemical and pharmacological analyses in order to give scientific ground to the ethnomedicinal knowledge.

## Background

Knowledge of the medicinal plants of Ethiopia and their uses provide wide and vital contribution to human and livestock healthcare needs throughout the country [1-5]. These wide and vital uses of traditional medicine in the country could be attributed to cultural diversity and acceptability, psychological comfort, economic affordability, and perceived efficacy against certain type of diseases as compared to modern medicines [6,7]. In Ethiopia, about 80% of the human population and 90% of livestock is said to be dependent on traditional medicine for primary healthcare services and most of this comes from plants [8,9]. That is why there are considerable number of research works on the various aspects on traditional medicinal plants [2,5-7,9-17] even some were developed to the pharmaceutical industries like, *Phytolacca dodecandra* L’Herit. [18,19]. However, many more medicinal plants of Ethiopia which are found in lesser studied areas still anticipate scientific studies.

The reviewed literatures show that studies on medicinal plants of Ethiopia have so far concentrated in the south, southwest, central, north and north-western parts of the country [2,5-7,9-13,15,16,20-33]. There were little data that quantitatively assess the resource potential, indigenous knowledge on the use and management of medicinal plant species from eastern Ethiopia [34,35] as well as none are there from the present study area.

The Oromo people who currently inhabit the prehistoric Harla and the entire catchments might be the descendents of the former Harla people of the Harla kingdom which had been ruled between 13^th^ to 16^th^ centuries (Patacini D, Berehanu K: Notes on Harla: a preliminary report, Unpublished). They are expected to be the guardians of valuable indigenous knowledge on the use of traditional medicinal plants of their surroundings, which they use for treating human and livestock ailments. Scientific investigations indicated that there is an endemic plant species named after this prehistoric place called *Aloe harlana* Reynolds [36] due to its availability only in Harla locality. It has been traditionally used by the Oromo people in Harla for the treatment of various infectious and inflammatory diseases [17]. The latex and isolated compounds of *A. harlana* possess promising antimicrobial activity particularly against the Gram-negative bacterial strains such as *Escherichia coli*, *Salmonella typhi* and *Vibrio cholerae*[17]. Unpublished documents suggested that there are many more potential medicinal plants in this unique geographic setting and complex landscape areas.

Even one of the translations of the eastern port town of Ethiopia known as Dire Dawa is “plain of medicine” in Oromo language. Dire Dawa is only 15 kms far from Harla and this study also covers 5 to 25 kms distant areas from this village believing that most of the traditional medicinal plants which are marketable in Dire Dawa are coming from these study areas (Harla and Dengego valleys and the entire catchments of Dire Dawa). In addition, given the diversity of plant species in the Dengego Mountains and valley complex, and the ancient history and civilization of the vanished Harla Kingdom, the share of medicinal plants and the value of the associated indigenous knowledge of the current Oromo communities of the area, who might be descendents of the lost Harla people, is expected to be high.

However, except few archaeological studies [23,37], this prehistoric place and people, Dire Dawa and entire Dengego mountain and valley complex are ethnobotanically unexplored and there is no comprehensive account of the traditional medicinal practices. Therefore, the objectives of this study were to (1) assess, identify and document the traditional medicinal plant species potential; (2) investigate comprehensive information on associated indigenous knowledge of medicinal plants; (3) generate baseline ethnomedicinal information on medicinal plants for human ailments for further investigation. Thus, the output of this study can be used as a steppingstone for conservation of medicinal plant species, preservation of ethnomedicinal knowledge, and phytochemical and pharmacological analysis.

## Methods

### Study area

The study area covers Harla upto Biyo Awale and Dengego Mountains and Valleys complex which is found under Dire Dawa administrative council. It extends 5 to 25 kms distance SE of Dire Dawa town in eastern Ethiopia which is located at 515 kms east of Addis Ababa and 311kms west of Djibouti.

**Figure 1:**
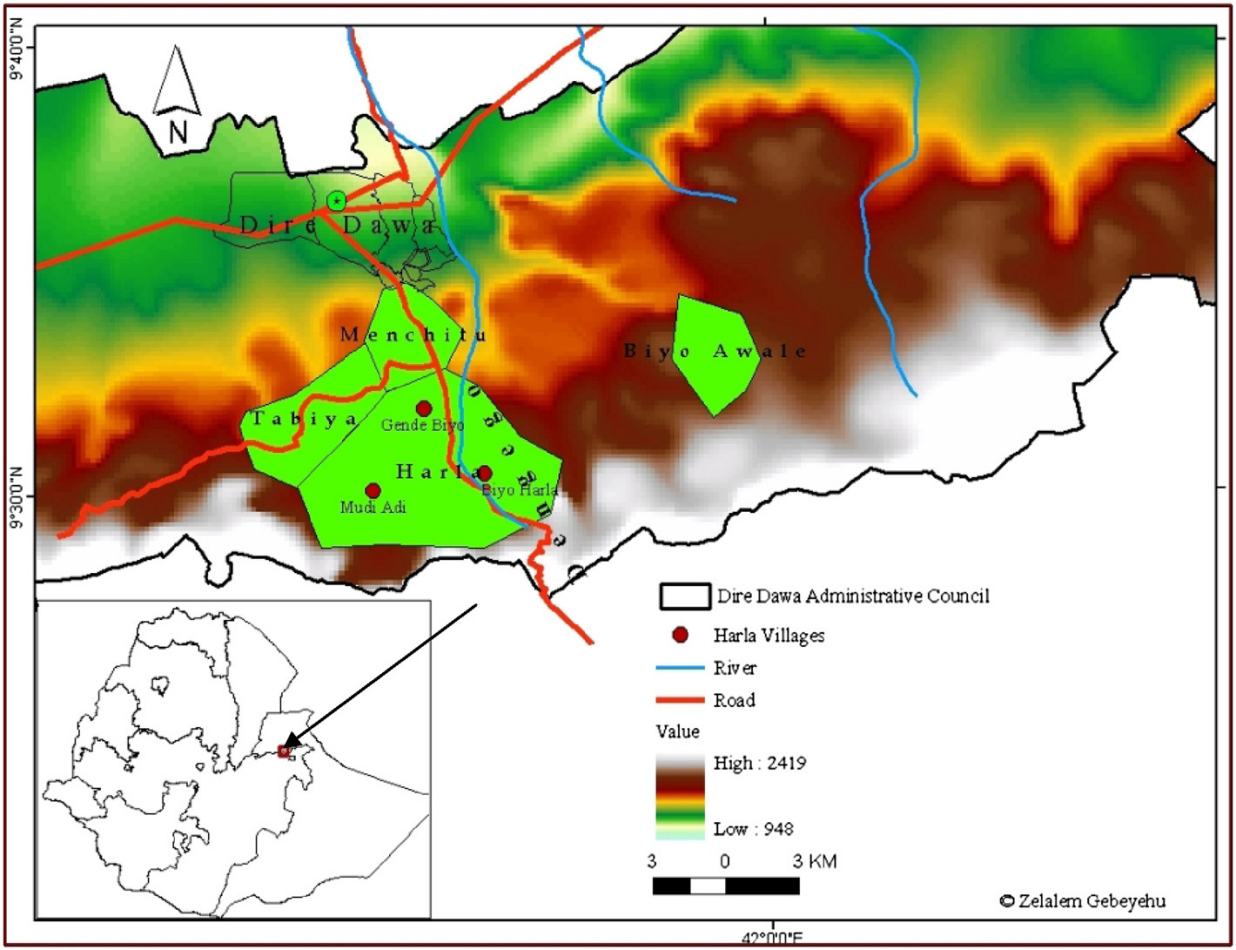
Map of study area indicating the prehistoric Harla with neighbouring kebeles and Dengego mountains/valleys complex.

This area is delimited with coordinates of 9°27′ and 9°39′N latitude and 41°38′ and 42°20′E longitude. Its elevation ranges between 950–2260 meters a.s.l. (Figure 1). The physiographic feature includes mountain ranges, hills, valleys, river terraces and flat plains. The geology of the area consists of precambrian metamorphic rocks (Gneisses, pegmatites and diorites), mesozoic sedimentary rocks (Adigrat sandstone, Hamanlei limestone and Amba Aradam sandstone), Tertiary volcanic (basalts) and quaternary sediments (alluvial sediments, travertine and river sand deposits) [38].

The mean annual temperature is about 22.8°C, ranging from a mean minimum of 16.2°C to mean maximum of 30.4°C. May to June are the hottest months of the area; whereas, November to January are the coldest months. The mean annual rainfall in the surrounding areas ranges from about 1,000 mm on the south to about 500 to 600 mm in the north lowland. Almost all of the catchments receive less than 900 mm year^-1^ of rainfall. Rainfall is bimodal, occurring from February to April (short rainy season) and June to September (long rainy season). The mean annual runoff values estimated for different watersheds ranges from 12.4 Mm^3^ to 100.13 Mm^3^[39].

The human population of the rural area is about 125, 800 (Male 63,000 and 62,800 female) in which the livelihood depends mainly on smallholder agriculture and livestock production [38]. The vegetation of the area includes few grass lands and wood lands, scrubland and bush lands dominated by species like *Acacia brevispica* Harms, *A. bussei* Harms ex Sjostedt, *A. etbaica* Schweinf, *A. seyal* Del*., Aloe megalacantha* Baker, *A. harlana* Reynolds*, Balanites aegyptiaca* (L.) Del. *Euclea racemosa* Murr., *Euphorbia bergeri* M. Gilbert, *Ficus salicifolia* Vahl. *Opuntia ficus-indica* (L.) Miller*,* and *O. stricta* Haworth*.*

Harla is probably a 13^th^ C village. As the finding of the site indicated, it has a long time commercial link with the middle and Far East through the port of Zeila between 13^th^ and 16^th^ C (Patacini D, Berehanu K: Notes on Harla: a preliminary report, Unpublished). The whole village was buried beneath the surface and covered with ashes and pumice. The current Harla is built on top of the old one. The current inhabitants use ready rectangular stone blocks from the old village, which they have uncovered while digging below the surface, to build their homes, fences, and farm land terraces (Pers. Observation and communication).

There are very limited written documents on the history of the Harla kingdom. Due to limitation of published works on the prehistoric Harla, we are unable to mention many references in this study except for a few indicated issues that may attract field professionals for future investigations. Of course, there are certain archaeological findings collected by different social anthropologists and archaeologists that are kept for visitors in the small museum at the centre of Harla village. Archaeological findings and collections of the site include coins written in Arabic and Chinese, pieces of glasses, ornaments, tools for knitting, pottery fragments and a stone moon calendar with two geographical coordinates, etc. which are available in some homes of the residents and in the small museum at the centre of Harla village (Pers. observation).

### Data collection methods

Participatory Rural Appraisal (PRA) techniques were employed to collect data, as recommended by Martin [40] and Cunningham [41]. Employing this methodology, an ethnobotanical data were collected in two different rounds, from October to December 2012 and May 2013 from six sampling sites which were identified from the study areas namely Biyo Harla, Gende Biyo, Mudi Adi, Tabiya, Menchitu and Biyo Awale. The first three sites were villages in Harla kebele and the other three sites were purposively selected as neighbouring kebeles (the smallest political administrative unit in Ethiopia) of the prehistoric Harla to represent Dengego valley complex. Ethnobotanical information was collected from 55 informants (44 male and 11 female). Among the 55 informants, 10 key informants (traditional healers) were selected with the assistance of community leaders, elderly people and members of the local community. Purposive sampling technique was used for selecting key informants (all were male and above 55 age) while stratified random sampling was employed to select others (34 male and 11 female). Households of selected study sites were registered and stratified into three age groups. Then fifteen informants were randomly selected from each stratum (age group) to see how the knowledge varies with age. The three age groups were young (25–40), adult (41–60) and elderly (above 60).

Before carrying out the interviews and group discussions, a traditional ceremonial and blessing of the Oromo culture conducted and an oral Prior Informed Consent (PIC) was confirmed from every respondent. Furthermore, participants collectively endorsed the research by giving oral blessings in their usual traditional style. Semi-structured interviews with 55 informants and group discussions (total of 9 groups discussed with average members of 11 per group) were administered in the local language (Afan Oromo) to collect basic information on the local name(s) and traditional description of the medicinal plant species, diseases treated or controlled, parts used, conditions and method of preparations, routes of remedial administration, dosages used, major drawbacks, and locally marketable medicinal plants. Besides, practical observation sessions in preparation of remedies and some observation of traditional treatment given to the patients by traditional healers were conducted. In addition, guided field walks with key informants were employed to collect voucher specimens of each medicinal plant species with additional notes. Photographic cameras were used for graphic documentation. Additional interviews with key informants were carried out in the field in order to avoid the risk of confusing identity of plant species by repeated inquiries. This was done for at least three times with the same and different informants so as to confirm the validity and reliability of the recorded information. Specimens were collected and numbered on the spot, later identified using taxonomic keys in the relevant volumes of the Flora of Ethiopia and Eritrea and through visual comparisons with authenticated plant specimens kept at the Herbarium of Haramaya University (HHU) where voucher specimens of the medicinal plants were deposited. The authentication of identified plant species was done by a renowned plant taxonomist, Mr. Melaku Wondafrash (National Herbarium of Addis Ababa University).

### Data analysis

The data were filled in Excel sheet in a way that makes the analysis very suitable. Total number of traditional medicinal plant species used for human ailments along with their Family and genus distribution; growth habit in percentage; part used versus number of remedies prepared; number of human ailments treated; methods of preparation, and route of administration were all analyzed using both qualitative and quantitative methods following Martin [40] and Cotton [42]. The informant consensus factor (*Fic*) of each medicinal plant, the proportion of informants who independently reported its use against a particular disease/disease category, was calculated using the formula: *Fic* = *n*_*ur*_–*n*_*t*_/*n*_*ur*_–1 [43,44], where, *n*_*ur*_ is the “number of use-reports” in each disease category and *n*_*t*_ is the “number of taxa used”. The *Fic* values range from 0 to 1, with high values (i.e. close and equal to 1) indicating that relatively few plants are used by a large proportion of informants, while low values (<0.5) indicate that informants do not agree on the plant species to be used to treat a category of ailments.

Frequency of citation (F) of each medicinal plant species was calculated using the formula:

$$\begin{array}{ll} F\;\left(\%\right)= & \mathrm{No}.\;\mathrm{of}\;\mathrm{informants}\;\mathrm{who}\;\mathrm{cites}\;\mathrm{the}\;\mathrm{species}/\\ & \mathrm{Total}\;\mathrm{No}.\;\mathrm{of}\;\mathrm{informants}\times 100. \end{array}$$

Binomial test was run in SPSS 18.0 to evaluate the depth of knowledge with age categories in which pair wise age category test was considered and for comparison of gender wise depth of knowledge. P-value of less than 0.05 was taken as statistically significant difference. MS Excel Spreadsheet was used to generate bar graphs.

## Results and discussion

### Medicinal plant species richness and part used for remedial preparations

This study revealed that the prehistoric Harla and Dengego Mountains and Valleys complex harbour about 83 traditional medicinal plant species against 81 human ailments which are distributed across 70 genera and 40 Families (Table 1). About 57.8% of these traditional medicinal plant species belong to ten Families. Asteraceae had the largest number of plant species (10, 12%), followed by Fabaceae (8, 9.6%), Euphorbiaceae (6, 7.2%) and Cucurbitaceae (5, 6%). Aloaceae and Lamiaceae had each 4 plant species, Asclepiadaceae, Boraginaceae and Capparidaceae each has 3 species, and Apocynaceae has 2 species. About 71% of these medicinal plant species were reported by different authors who conducted researches on traditional medicinal plants in the different parts of Ethiopia [4-7,9-13,15,16,21,22,24,25],[27-32,34,45] wherein about 44% of them were reported for similar ailments. The number of medicinal plant species reported in this study is considerable, though application of long-term participant observation techniques could add more medicinal plant species to the present list, given the floristic richness and the strongly plant-based bio-cultural background of the people. In addition, there is a potential market of traditional medicine in the nearby towns like Dire Dawa stretching to Djibouti. That is why few traditional practitioners were reluctant to give all the information since this could be detrimental to the economic benefits that come out of the traditional medicine. So, the economic benefits coming out of the traditional medicine restricted the information to some extent.

**Table 1:** List of traditional medicinal plant species used to treat human ailments in the prehistoric Harla and Dengego valleys

**Voucher No.**	**Scientific name**	**Family**	**Vernacular name**	**Habit**	**Disease treated**	**PU**	**Method of preparation & part administered**
AHU167	*Abutilon bidentatum*	Malvaceae	Muka Adi	HA	Headache	L	Boiled in hoja and served like a tea
	(Hochst.) A. Rich.				Rh disease	L	Decoction taken orally
					Mineral deficiency in children	L	Infusion taken oral
AHU212	*Acacia nilotica* (L.)	Fabaceae	Serkema	T	Bad breath/halitosis	B	Chew and spit
	Willd. ex Del.						
AHU197	*Acalypha fruticosa*	Euphorbiaceae	Dhirii	Sh	Heart disease	L	Decoction is taken oral
	Forssk.				Kidney infection/Nephropathy	L	Decoction is taken oral
AHU171	*Acanthospermum*	Asteraceae	Kumutu Adi	HA	Itching skin	Ap	Concoction is drenched
	*hispidum* DC.						
AHU33	*Acokanthera schimperi*	Appocynaceae	Qarari	Sh	Tonsillitis	St, L	Concocted, gargling and rinsing the throat
	(A. DC.) Schweinf.				Malaria	Ap	Dried & smoke is used as mosquito repellent
AHU117	*Aloe harlana Reynolds	Aloaceae	Hargesa	Sh	Snake bite, liver swelling & spleen swelling/Splenomegaly	L	Crushed and filtrate taken oral in all cases
					Colon cleaner	Sa	Crystallized, powdered and juice taken oral
					Skin fungus, hair fungus & skin inflammation	J, L	Concocted together and used as ointment and wash the hair
						Sa, J	
AHU161	*Aloe mcloughlinii Chris.	Aloaceae	Hargesa	Sh	Eye infections	Sa	Extract the sap and drop in the eye
AHU162	*Aloe megalacantha* Baker	Aloaceae	Hargesa Guracha	Sh	Colon cleaner	J, Sa	Crystallized & Juice made/sibri, taken oral
AHU160	*Aloe retrospiciens*	Aloaceae	Hargesa Adi	Sh	Colon cleaner	J, Sa	Crystallized & Juice made/sibri, taken oral
	Reynolds & Bally						
AHU153	*Asparagus africanus* Lam.	Asparagaceae	Hida Sere	Sh	Swelling and infection on the head (korokor)	L	Crushed and put on hot plate and applied on the head while warm
AHU201	*Asparagus racemosus* Willd.	Asparagaceae	Hida Sero Guracha	Sh	Body burning feeling and mentally disturbed	Br	Concocted and taken oral and drenched
					Itching the whole skin	R	Crushed and the filtrate is drenched
AHU213	*Azadirachta indica* A. Juss.	Meliaceae	Kinina	T	Malaria	S, L	Mixture of leaf infusion and oil extracted from seed taken oral
					Intestinal parasites	S, L	Mixture of leaf infusion and oil extracted from seed taken oral as anthelmintics
AHU207	*Bidens pilosa* L.	Asteraceae	Xiye	HA	Difficulty of blood clotting	St, L	Crushed and bandaged on bleeding part
AHU112	*Cadaba rotundifolia* Forssk.	Capparidaceae	Delensisa	Sh	Extended flow of menstruation/Menometrorrhagia	B, L	Concocted together with *Withania somnifera* and a cup of filtrate is taken oral
AHU178	*Cadia purpurea* (Picc.) Ait.	Fabaceae	Cheeka	Sh	Gastritis	N	Collect from the flower and taken oral
					Heartburn/Pyrosis	N	Sucked from flower and used as carminative
AHU146	*Capparis tomentosa* Lam.	Capparidaceae	Gemora	CP	Nipple pores remain closed after birth	R, L	Concoction taken oral to facilitate opening of nipple pores
AHU111	*Caralluma speciosa* N.E. Br.	Asclepiadaceae	Ya’ii Bera	HP	Skin cyst & tumour locally known as keledo	St	Crushed with *Gloriosa superba* and put on the tumour
					Gangrene	St	Powdered with *Gloriosa superba* and turtle bone then put on the starting point
					Swollen body part-gofla	St	Crushed and bandage on swollen part
					Anti poison	Sa	Diluted sap taken orally
					Wound	Sa	Sap extracted and used as ointment
					Itching skin	Sa	Sap extracted and used as ointment
AHU154	*Carissa spinarum* L.	Apocynaceae	Agamsa	Sh	Premature ejaculation	F	Decoction of unripened fruit is served as a tea and wash the body with the infusion
AHU199	*Cissampelos mucronata* A. Rich.	Menispermaceae	Bal-Toke	CP	Sudden illness locally called dingetegna	R	Chew & swallow to stop sudden vomiting, abdominal pain and discomfort
AHU204	*Coccinia sp*. Burger	Cucurbitaceae	Hanchota	CH	Kidney disease	Tu	Infusion taken oral
AHU214	*Commelina stephaniniana* Chiov.	Commelinaceae	Hola gebis	HA	Skin fungus around the neck and face	Sa	Extract creamy sap and use as an ointment
AHU187	*Commicarpus sinuatus*Meikle	Nyctaginaceae	Kontom	HP	Gonorrhoea	L	Concoction with leaf and fruit of *Cucumis dipsaceus* and taken oral
					Skin fungus around the neck and face	L	Leaf paste mixed with oil and used as ointment
AHU184	*Craterostigma plantagineum* Hochst.	Scrophulariaceae	Roba Enjire	HA	Liver disease	R, L	Concoction taken oral
					Diarrhoea	R, L	Concoction taken oral
AHU158	*Croton macrostachyus* Del.	Euphorbiaceae	Bekenisa	T	Liver disease/Jaundice	B	Concocted with bark of *Terminalia brownii* and drink a cup of infusion
AHU108	*Cucumis dipsaceus* Ehrenb. ex Spach	Cucurbitaceae	Hare Goge	CH	Gonorrhoea	F, L	Concocted with *Commicarpus sinuatus* leaf and taken oral
					Urinary retention/Ischuria	L	Crushed and filtrate taken oral
					Skin fungus	F	Rub the affected part with warm fruit
AHU108B	*Cucumis ficifolius* A. Rich.	Cucurbitaceae	Hare Goge	CH	Swelling due to poisonousthorn	F	Put on hot plate and bandage on the swollen part while warm
AHU217	*Cucumis prophetarum* L.	Cucurbitaceae	Hidi	CA	Wound and Swollen body part	F	Make it warm and bandage on wound/ swollen part while warmth
AHU114	*Cynoglossum coeruleum* Hochst. ex A. DC.	Boraginaceae	Mexene Tiro	HA	Kwashiorkor	Ap	Concocted with *Verbascum sinaiticum* and taken oral
AHU149	*Datura stramonium* L.	Solanaceae	Qamaxari	HA	Ear infections/Otitis externa & media	F, L	Dried, ground together and mix with oil and drop in the ear
					Worms created in the tooth gum	F	Boiled and put on the gum area
AHU159	*Dodonaea angustifolia* L.f.	Sapindaceae	Edecha	Sh	Hair fungus Swelling and bursting on the head (korokor)	L	Dried, powdered and mixed with oil and used as an ointment
					Malaria	F	Fresh fruits are eaten
					Intestinal parasite	L	Fresh leaf extract taken oral as anthelmintics
AHU164	*Echidnopsis dammanniana* Spren.	Asclepiadaceae	Muka Mesqa	HA	Snake bite poison	St	Crushed and tie on the snake bite
AHU209	*Echinops macrochaetus* Fresen.	Asteraceae	Qore Hare	HA	Toothache	R	Crushed and put on the painful teeth
AHU196	*Erianthemum aethiopicum* Wiens & Polhill	Loranthaceae	Digelo Serkema	E	Breast swelling/Mastitis	St, L	Concoction taken oral
AHU192	*Erucastrum arabicum* Fisch. & Mey.	Brassicaceae	Rafu Shimbiro	HA	Skin fungus around the neck and face	P, S	Dried, powdered, mix with oil and use as an ointment
AHU118	*Euclea racemosa* Murr. subsp. s*chimperi*	Ebenaceae	Miesa	Sh	Snake bite poison	L	Crushed with *Aloe sp*. and filtrate taken oral
					Liver swelling	L	Crushed with *Aloe sp.* and filtrate taken oral
					Spleen swelling/Splenomegaly	L	Crushed with *Aloe sp.* and filtrate taken oral
AHU163	*Eulophia petersii* Rchb.f	Orchidaceae	Shunkurta Gara, Ejiji	HA	Swollen body part-gofla	Bu	Cooked bulb eaten
					Abdominal pain/kurtet	Bu	Soup made from bulb taken oral
AHU110	*Gloriosa superba* L.	Colchicaceae	Harmel Kubra	Sh	Toothache	L	Crushed leaf applied on painful teeth
					Epilepsy	L	Crushed leaf filtrate taken oral
					Skin cyst & tumour/keledo	L	Crushed leaf is bandage on tumour/cyst
					Gallstone	L	Immersed in water and infusion taken oral
					Gangrene	L	Crushed with succulent *Caralluma speciosa* and tie on the starting point
AHU126	*Gomphocarpus purpurascens A. Rich.	Asclepiadaceae	Ari-Yuyo	HA	Itching skin	L	Roasted and powdered leaf is mixed with oil & used as ointment
					Evil eye	L	A cup of Infusion taken oral & smoke bath with dry leaf
AHU145	*Gossypium hirsutum* L.	Malvaceae	Jibri Boke	Sh	Small swelling with oozing pus in the vagina/Vaginitis	L	Concocted with *Acokanthera schimperi* and kurunfud and wash the affected part
AHU177	*Grewia bicolor* Juss.	Tiliaceae	Deka	Sh	Small swelling with oozing pus/skin ulcer	L	Crushed leaf in bandage on it
					Epidermal drying	L	Extract is applied on skin as emollients
					Bad breath (Halitosis)	St	Used as a toothbrush
AHU144	*Heliotropium aegyptiacum* Lehm.	Boraginaceae	Harma Deysa	HA	Leech attached on throat	L	Crushed and filtrate is used for gargling the throat
AHU166	*Heliotropium steudneri* Vatke	Boraginaceae	Muka Michii	HA	mich	L	Crushed and filtrate is drenched
					Skin fungus	L	Fresh leaf rubbed on affected part
AHU169	*Indigofera amorphoides* Jaub. & Spach	Fabaceae	Muka Adi	HP	Heart disease	Ap	Decoction taken oral
AHU176	*Indigofera* sp.	Fabaceae	Muka Aroo	HA	Herpes zoster	L	Dried, powdered, roasted and mixed with oil to be used as ointment
AHU174	*Indigofera ellenbeckii Bak. f.	Fabaceae	War	HP	Mouth infection	L	Crushed leaf filtrate is used to wash mouth
AHU189	*Jasminum grandiflorum* L.	Oleaceae	Bilu	Sh	Chapped lips	L	Paste of fresh leaf used as emollient on lips
					Tooth gum infection/Gingivitis	L	Crushed and applied on the gum in the mouth
AHU152	*Jatropha curcas* L.	Euphorbiaceae	Hambete Muluk	Sh	Constipation	S	Decocted and oily fluid taken oral as laxative
AHU203	*Kalanchoe marmorata* Bak.	Crassulaceae	Chophi Gurati	HP	Eye infection	St	Extracted sap is boiled, cooled & dropped
					Ear infections/Otitis	St	Sap extracted, boiled, cooled & dropped
					Swelling with pus due to spine	St, L	Crushed with *Ricinus communis* seed and bandage on to remove the pus and spine
AHU200	*Kleinia abyssinica* (A. Rich.) A. Berger	Asteraceae	Abrasha	HA	Sexual dysfunction	Rh	Aphrodisiac fresh rhizome is eaten few hours before sexual performance
AHU202	*Kleinia odora* (Forssk.) DC.	Asteraceae	Luko	HP	Nerve case	L	Oily extract is boiled, mixed with *Cadaba rotundifolia* and used to massage
AHU205	*Kleinia pendula* (Forssk.) DC.	Asteraceae	Afrasha	HP	Swollen body part	St	Decoction of fresh succulent is bandaged on swollen part while warm
AHU206	*Kleinia squarrosa* Cufod.	Asteraceae	Luko	Sh	Intestinal parasite	St	Crush and taken oral as anthelmintics
					Swelling on gum and toothache	St	Used as toothbrush
AHU195	*Lagenaria siceraria* (Molina) Standl.	Cucurbitaceae	Buqee	CA	Obstructed labour/Dystocia	L	Crushed and filtrate taken oral in a traditional assisted delivery
AHU173	*Leucas minimifolia* Chiov.	Lamiaceae	Muka Adi	Sh	Eye diseases	L	Crushed and filtrate dropped in the eye
					Closing of the eye in the morning specially children	L	Crushed and filtrate dropped in the eye
AHU175	*Leucas stachydiformis (Hochst. ex Benth.) Briq.	Lamiaceae	Muka Bofta	HA	Mouth infection	L	Decoction taken oral
					Nose infection	L	Decoction taken oral
AHU150	*Lawsonia inermis* L.	Lytheraceae	Hina	Sh	Infection after haemorrhage & skin tumour removal	L	Crushed fresh leaf is applied external as antiseptic
						L	
					Fever	L	Crushed and wash the head
AHU148	*Maerua triphylla* A. Rich.	Capparidaceae	Qanqalcha	Sh	Stomach gofla	L	Concocted and taken oral
AHU127	*Melhania zavattarii* Cufod.	Sterculiaceae	Muka bira	Sh	Kidney infection	F, L	Concoction taken oral
AHU179	*Ocimum basilicum* L. var. *thyrsiflorum* (L.) Benth.	Lamiaceae	Rahan	HP	sirkita	L	Crushed and filtrate taken oral
AHU140	*Ocimum lamiifolium* Hochst. ex Benth.	Lamiaceae	Rahana, Riroo	Sh	mich	L	Crush and squeeze the solution to drench and drink a teaspoon of it with tea or coffee
					Eye infection	L	Immerse in water and wash the eye with diluted infusion
AHU215	*Opuntia ficus-indica* (L.) Miller	Cactaceae	Tini	Sh	Hair fungus	J	Extracted and wash the hair
AHU210	*Osyris quadripartita* Decn.	Santalaceae	Wato	Sh	Malaria	R, L	Reddish infusion resulted after 24 hours immersion is taken oral
AHU105	*Phyllanthus maderaspatensis* L.	Euphorbiaceae	Harmel Xixiqaa	HA	Heart disease	Ap	Concocted and taken oral
AHU147	*Plumbago zeylanica* L.	Plumbaginaceae	Merxes	HA	Low level swelling under skin	R, L	Decoction taken oral
AHU216	*Portulaca oleracea* L. subsp. *oleracea*	Portulacaceae	Merere Haree	HA	Constipation	L	Cooked and served as laxative vegetable
					Cough	L	Cooked and eaten as a demulcent agent
AHU142	*Pouzolzia parasitica* (Forssk.) Schweinf.	Urticaceae	Dirba	HA	Infertility in female	R, L	Concoction taken oral to increase the chance of fertility
AHU142	*Pouzolzia parasitica* (Forssk.) Schweinf.	Urticaceae	Dirba	HA	Diarrhoea	Tu, L	Crushed together and infusion taken oral
					Haemorrhage	L	Washing of anal opening with the infusion
					Hair fungus	Tu, L	Concocted to wash the hair
AHU151	*Prunus persica* (L.) Batsch	Rosaceae	Kuki	T	Snake bite poison	L	Crushed and filtrate taken oral
					Liver swelling	L	Infusion taken oral
					Spleen swelling/Splenomegaly	L	Infusion taken oral
AHU208	*Punica granatum* L.	Lythraceae	Roman (Am)	T	Swollen body part/gofla	F	Decoction serves like a tea
AHU172	*Pupalia lappacea* (L.) A. Juss.	Amaranthaceae	Metene	HA	Urinary retention/Ischuria	Ap	Concoction taken oral
AHU194	*Reichardia tingitana* (L.) Roth	Asteraceae	Wachara Haree	HA	Liver disease/swollen and create fluid sacs	L	Decoction with sugar taken like a tea
AHU109	*Rhynchosia erlangeri Harms	Fabaceae	Harmel	Sh	Mental problem	L	Crushed leaf filtrate taken oral
					Heart disease	L	Concocted mix with honey and taken oral
AHU119	*Ricinus communis* L.	Euphorbiaceae	Qobo	T	Constipation	S	Extracted oil taken as oral laxative
AHU182	*Senna italica* Mill.	Fabaceae	Tenemeki	HA	Colon cleaner	L	Concocted with the fruit of *Tamarindus indica* and sugar and used as laxative
AHU181	*Steganotaenia araliacea* Hochst. ex A. Rich.	Apiaceae	Harfetu	T	Body burning feeling and mentally disturbed	L	Concocted with *Grewia sp*. and *Cissampelos mucronata* and taken oral
AHU183	*Tamarindus indica* L.	Fabaceae	Roka	T	Colon cleaner	F	Concocted with *Senna italica* and sugar and used as laxative
AHU155	*Terminalia brownii* \Fresen.	Combretaceae	Bireysa	T	Liver disease/Jaundice	B	Concocted with bark of *Croton macrostachyus* and drink a cup of infusion
AHU157	*Tragia plukenetii* A. Rodel.-Smith	Euphorbiaceae	Dobi	HA	Nipple opening remain closed after birth	R, L	Concocted and filtrate is taken oral
AHU185	*Tribulus terrestris* L.	Zygophyllaceae	Qumutu Gala	HA	Heart disease	Ap	Concoction taken oral
AHU143	*Verbascum sinaiticum* Benth.	Scrophulariaceae	Muka loni, Gura Haree	Sh	Kwashiorkor	Ap	Concocted with *Cynoglossum coeruleum* and taken oral
AHU156	*Vernonia amygdalina* Del.	Asteraceae	Ebicha	T	Liver disease/Jaundice	B	Immerse in water and drink the infusion
AHU141	*Withania somnifera* (L.) Dun. in DC.	Solanaceae	Hidi Bude	Sh	Extended flow of menstruation/Menometrorrhagia	B, L	Concocted together with *Cadaba rotundifolia* & a cup of filtrate is taken oral
					Gallstone	R, L	Concoction taken oral
					Evil eye	Br	Dried and smoke bath
AHU188	*Zinnia peruviana* (L.) L.	Asteraceae	Muka Ilili	HA	Depigmentation of section of skin/Vitiligo	R, L	Concocted and applied on affected part
AHU104	*Ziziphus spina-christi* (L.) Desf.	Rhaminaceae	Kurkura	T	Haemorrhage	L	Crushed and bath the anal opening
					Headache	L	Concocted and used to wash the head

**Habit**: Sh-shrub, T-tree, CA-climber/annual, CP-climber/perennial, HA-herb/annual, HP-herb/perennial, E-Epiphyte, CH– creeper herb, Part Used (**PU**), AP-All parts, B-bark, Br-branches, Bu-Bulb, St-stem, Sa-sap, J-jel, R-root, F-fruit, L-leaf, S-seed, Tu-tuber, P-Pod, Rh-rhizome and N-nectar.

*Endemic species.

Among the medicinal plants identified in this study, various parts of 12 medicinal plants species were reported to be sold in the open markets of the nearby towns like Dire Dawa. The dried and powdered sap of *Aloe harlana* and *A. megalacantha,* seeds and leaves of *Azadirachta indica*, leaves of *Gloriosa superba* and *Lawsonia inermis;* and the fresh root of *Cissampelos mucronata,* tuber of *Coccinia sp.,* leaf of *Ocimum lamiifolium,* fruits of *Punica granatum* and *Tamarindus indica* were sold in the open local market places. Similarly, ample domestic trade of Ethiopian medicinal plants was reported for diverse cultural groups in Ethiopia [6,7,16,21,23,26,34]. In contrary, none of the medicinal plants reported by some other studies were available for sale in local markets [2,5,10]. These might be related with the norm and cultural issues of diverse communities who permit and prohibit marketing of traditional medicines.

Analysis of the growth habits of the traditional medicinal plant species showed that shrubs constitute the highest number of species and epiphytes the least number of species, represented by only one species (*Erianthemum aethiopicum*/Loranthaceae) (Table 2).

**Table 2:** The number of traditional medicinal plant species in each growth habit

**Growth form**	**No. of species**	**Percentage**
Shrub	29	34.9
Herb/annual	28	33.7
Tree	11	13.3
Herb/perennial	7	8.4
Creeper/herbaceous	3	3.6
Climber/annual	2	2.4
Climber/perennial	2	2.4
Epiphyte	1	1.2

The highest proportion of growth habit was covered by shrubs and herbs that constitute 68% of the total traditional medicinal plants. This can be related to the floristic composition of vegetation, which is dominated by woodland, bushland and scrubland vegetation types both in valleys and rocky mountains. Similar patterns were reported by some medicinal plant inventories work [16,24,25] where shrubs and herbs are the largest plant growth habits but contrary to some works [6,34] where woody plant species dominated the growth form.

**Figure 2:**
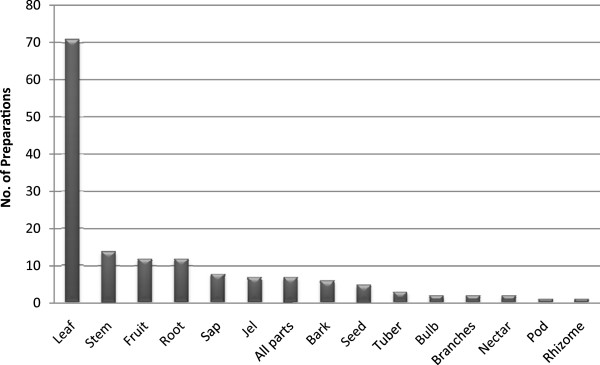
The use of different plant parts in remedial preparation and number of preparations per plant part.

A total of 15 different parts of the medicinal plant species are used for remedies preparation (Figure 2). Remedial preparations made from leaves accounted for 46.4%, stem 9.2%, fruits and roots each 7.8% of the total preparations. This could be a large number of plant parts used in remedial preparation when compared with the various research reports done on traditional medicinal plants [15,21,22,28-30,34]. Such diversified use of plant parts in remedial preparation could be considered as an indicator of the deep rooted and long lasting practice and know-how of traditional medicinal plants by the community.

A total of 140 preparations were made using these 15 different parts of the medicinal plant species. The most frequently sought parts of the medicinal plant species were leaf, fruit, seed, branches, pod, and nectar that account for 60%. This may lead to the conclusion that harvesting medicinal plants poses no significant threat to the natural vegetation of the study area. Similarly, in studies conducted elsewhere in Ethiopia, leaf was indicated to be the most frequently used plant part in remedial preparations that do not cause any significant threat to the survival of individual plants when compared to other plant parts such as underground part, stem, bark and whole plant [2,15,16,34,46]. In contrast, other studies [30-32] indicated root and bark as the most commonly harvested plant part for remedial preparations. For example, study conducted in Benshangul-Gumuz of Ethiopia [30] reported that about 63% of the preparations were made from root and bark of medicinal plants. It is a mere fact that medicinal plants that are harvested for their roots, rhizomes, bulbs, bark, stem and whole part have severe effects on their survival [1,3] but this could be more important for the perennial and woody plant species.

### Informant consensus factor and frequency of citation

The most common health problems of the population of the study area were identified by traditional healers based on their experience on frequency of ailments treatment. In this respect, a total of 11 ailments were reported as the most common health problem of the study area. The *Fic* value for these most important health problems of the area ranges between 0.77 and 1 (Table 3). The *Fic* is higher for gastritis and heartburn/pyrosis (1.0) and relatively lower for swollen body part locally called gofla (0.77). The *Fic* results could be useful in prioritizing medicinal plant species for further pharmacological studies [10,25] since efficacy of traditional medicinal plant is strongly correlated with *Fic* value, meaning pharmacologically effective remedies are expected to have greater *Fic* value and vise versa [43].

**Table 3:** Major types of human health problems of the study area, number of plant species used and informant consensus factor values

**Major health problems of the study areas**	**List of plant species used and no. of citation in the bracket**	**Total no. of use citation**	**Fic value**
Gastritis and heartburn/pyrosis	*Cadia purpurea* (8)	8	1
Constipation	*Jatropha curcas* (10), *Portulaca oleracea* (19), *Ricinus communis* (11)	40	0.95
Haemorrhage	*Pouzolzia parasitica* (11), *Ziziphus spina-christi* (7)	18	0.94
Intestinal parasite	*Azadirachta indica* (11), *Dodonaea angustifolia* (9), *Kleinia squarrosa* (3)	23	0.91
Skin cyst & tumor	*Caralluma speciosa* (3), *Gloriosa superba* (4), *Plumbago zeylanica* (15),	22	0.90
Diarrhoea	*Craterostigma plantagineum* (4), *Pouzolzia parasitica* (5)	9	0.88
Kidney infections	*Acalypha fruticosa* (4), *Coccinia sp.* (12), *Melhania zavattarii* (2)	18	0.88
Eye and ear	*Aloe mcloughlinii* (6), *Datura stramonium* (5), *Kalanchoe marmorata* (6), *Leucas minimifolia* (3), *Ocimum lamiifolium* (7)	27	0.81
Wound and external infections	*Asparagus africanus* (5), *Caralluma speciosa* (3), *Cucumis prophetarum* (7), *Dodonaea angustifolia* (4),*Gossypium hirsutum* (3), *Grewia bicolor* (3), *Jasminum grandiflorum* (5), *Kalanchoe marmorata* (4), *Leucas stachydiformis* (4), *Lawsonia inermis* (6)	44	0.79
Skin itching, fungus, inflammation	*Acanthospermum hispidum* (4), *Aloe harlana* (8), *Asparagus africanus* (3), *Caralluma speciosa* (3), *Commelina stephniniana* (5), *Commicarpus sinuatus* (4), *Cucumis dipsaceus* (7), *Dodonaea angustifolia* (2), *Erucastrum arabicum* (2), *Gomphocarpus purpurascens* (3), *Grewia bicolor* (3), *Heliotropium steudneri* (3), *Opuntia ficus-indica* (10), *Pouzolzia parasitica* (4)	61	0.78
Swollen body part locally called gofla	*Aloe harlana* (6), *Kleinia pendula* (3), *Caralluma speciosa* (3), *Cucumis ficifolius* (13), *Cucumis prophetarum* (4), *Euclea racemosa* (3), *Eulophia petersii* (5), *Erianthemum aethiopicum* (3), *Kleinia squarrosa* (3), *Maerua triphylla* (2), *Prunus persica* (2), *Punica granatum* (3), *Richardia tingitana* (4),	54	0.77

Even if the highest value of *Fic* was for gastritis and heartburn/pyrosis indicating that there is high consensus on the treatment of these major health problems of the area, it can be concluded that there are relatively high *Fic* values for their major health problems. This will attract pharmacologists for further pharmacological investigation of the traditional plant species in this rich ethnomedicinal knowledge and practice centre. The pharmacological study done in the prehistoric place of Harla by [17] indicated that the latex and isolated compounds of *A. harlana* possess promising antimicrobial activity particularly against the Gram-negative bacterial strains such as *Escherichia coli*, *Salmonella typhi* and *Vibrio cholerae*. Similar results were reported by [6,47] where the *Fic* value were greater than 0.5 for all clusters that may encourage interested researchers for validation of bioactivity as well as isolation and characterization of the active principles of those plant species in each category with high frequency of citation.

The frequencies of citation for medicinal plant species that are more popular and widely used by the local community were analyzed. Species having more than 20% frequency of citation are given in Table 4. A total of 18 plant species showed high frequency of citations ranging from 21.8–87.3 percent. *Aloe megalacantha* has the highest frequency of citation (87.3%) which was used as colon cleaner and a remedy made from it locally called sibri is sold in the local open market places, followed by *Cissampelos mucronata* (85.5%), *Aloe harlana* (78.2%), *Ocimum lamiifolium* (76.4), etc. (Table 4). This can show substantial level of agreement on the therapeutic worth of the traditional medicinal plant species in the study area. The greatest independent citations a particular species receives for treatment of a certain illness category is, the greatest its cultural importance [34].

**Table 4:** Plant species with the highest frequency of citation based on overall effectiveness to treat the corresponding human ailments

**Species name**	**Disease (s) treated**	**F** ***(%)***
*Aloe megalacantha*	Colon cleaner	87.3
*Cissampelos mucronata*	Sudden illness locally called “dingetegna”	85.5
*Aloe harlana*	Swollen body part locally called gofla, snake bite, liver swelling, spleen swelling, colon cleaner, skin fungus, hair fungus, skin inflammation	78.2
*Ocimum lamiifolium*	“mich”, eye infection	76.4
*Portulaca oleracea*	Constipation, cough	70.9
*Tamarindus indica*	Colon cleaner	70.1
*Withania somnifera*	Extended flow of menstruation/menometrorrhagia, gallstone, evil eye	69.1
*Cadia purpurea*	Gastritis, heartburn/pyrosis	67.3
*Azadirachta indica*	Intestinal parasite, malaria	56.4
*Lawsonia inermis*	Infection after hemorrhage removal, infection after skin tumor removal, fever	49.1
*Pouzolzia parasitica*	Diarrhoea, hemorrhage, hair fungus	47.3
*Kleinia abyssinica*	Sexual dysfunction	41.8
*Terminalia brownii*	Liver disease/jaundice	38.2
*Caralluma speciosa*	Skin cyst & tumor locally known as keledo, gangrene, swollen body part, anti poison, wound, itching skin	34.5
*Cucumis ficifolius*	Swollen body part locally called gofla	23.6
*Gomphocarpus Purpurascens*	Itching skin, evil eye	32.7
*Plumbago zeylanica*	Skin cyst & tumor locally called keledo	27.3
*Coccinia sp.*	Kidney infections	21.8

F = frequency of citation.

*Aloe harlana* was reported to be used for the highest number of ailments that treat swollen body part locally called gofla, anti-poison for snake bite, liver swelling, spleen swelling, colon cleaner, skin and hair fungus and skin inflammation. Another study [17] on *A. harlana* indicated that the Oromo people in Harla have been used it for the treatment of various infectious and inflammatory diseases. It has a considerable role in the primary healthcare system of the community. It is an endemic plant species known only in this study area and the specific epithet “*harlana*” refers to the prehistoric Harla, locality of type specimen. Until the time of this study, the community in Harla didn’t know that the famous and endemic traditional medicinal plant known as *A. harlana* is only found in their vicinity and nowhere else. Its sap extraction was dried, crystallized and powdered for the preparation of a popular traditional colon cleaner locally known as sibri (Oromo language), a product name on local market places. Indeed, this result will encourage local communities to further conserve and safeguard such valuable medicinal plant species within their ongoing wide scale conservation activities. A study conducted in Arsi zone of Ethiopia [29,33] indicated that paying special attention to high value medicinal plants could help to strengthen the role of those plant species in healthcare and environmental protection.

Plant species such as *Aloe megalacantha, Cissampelos mucronata, Ocimum lamiifolium, Tamarindus indica, Lawsonia inermis* and *Withania somnifera* scored high frequency of citations greater than 50 percent among the medicinal plant species which were marketable in the open market places. The higher frequency of citation of these species indicates their importance for local communities and attracts more attention for conservation in the study area.

The result on depth of comprehensive ethnomedicinal knowledge among different age groups indicated that elderly people (above 60 years) had much profound knowledge (binomial test, p = 0.002). Whereas, an ethnomedicinal knowledge test in the age group ranging from 25 to 40 showed the least value (binomial test, p = 0.008). There is a significant difference in the depth of ethnomedicinal knowledge between age category ranging from 25 to 40 and age category above 60 (p > 0.05). It was observed that many young people in the study area are less knowledgeable about the variety and value of indigenous medicinal plants. This might be attributed to the current expansion of education and health centres to kebele level which has resulted in the young generation focusing on modern medicines. Similar results were reported in some other cultural groups in Ethiopia [15,26] that showed the deterioration of indigenous knowledge on medicinal plants throughout the generations. A study conducted in Bale region of Ethiopia witnessed that western style health care services provided by government and NGOs seem to have contributed to a decline in traditional knowledge on medicine [28]. Therefore, documentation and communication of findings on knowledge and use of traditional medicinal plants in the present study area and beyond is very valuable in safeguarding the deterioration of indigenous knowledge on medicinal plants. Such findings need to be scaled-up followed by phytochemical and pharmacological analyses in order to give scientific ground to the ethnomedicinal knowledge.

In addition, the binomial test on ethnomedicinal knowledge between men and women showed that men have much more profound knowledge (binomial test, p = 0.001) than women (binomial test, p = 0.009) which is significantly different (p > 0.05). Similar results were reported by [9-11,34] where men have more profound knowledge than women in many parts of Ethiopia. This might be related with the local tradition of restricting traditional medical practices mostly to men and resulted in least number of women representation in the informant sampling of this stud. All the key informants (traditional healers) selected in this study were men, as it is also largely true for many other parts of Ethiopia. In contrast, [48] have reported women have more specialized knowledge on medicinal plants than men since they are often called upon to diagnose and treat certain types of diseases. It was also reported that men and women who are traditional medicine practitioners have relatively equivalent medicinal plants knowledge [26].

### Methods of preparation and routes of administration

The informants reported that 140 different preparations were made from 83 medicinal plant species. These were cited in the traditional healing system for use in 81 different human ailments. Out of the total preparations 50.7% are prepared in the form of concoction followed by fluids extraction (10.7%) and infusion (6.4%) (Table 5). Most of the remedies are prepared from a single species; mixtures are used infrequently. Out of total preparations, 127 were prepared from single plant species and the rest 13 were from two or more plant species. A number of sources [2,10,11,27,45,49] reported similar results stating that monotherapy preparation made from single plant species was used more frequently than mixtures for remedy preparations. This contrasts with the report by [9,15] where mixtures of different species were used to treat ailments than the use of single species.

**Table 5:** Method of preparation

**Method of preparation**	**Number of preparations**	**Percentage**
Concoction	71	50.7
Fluids extraction	15	10.7
Infusion	9	6.4
Crushed and pounded	8	5.7
Decoction	6	4.3
Ointment	5	3.6
Cooked as a soup	4	2.9
Dried and powdered	4	2.9
Make it warm/hot	4	2.9
Small cut of fresh part to be rubbed	3	2.1
Dried for smoke bath	2	1.4
Small cut of fresh part to be chewed	2	1.4
Small cut of fresh part to be eaten	2	1.4
Small cut of stick for brushing	2	1.4
Syrup	2	1.4
Pulverized and filtered	1	0.7

The preparations made from mixture of two plant species were like, the bark of *Croton macrostachyus* and *Terminalia brownii* were crushed, concocted and taken orally to treat jaundice. The stem and leaf of *Kalanchoe marmorata* and the seeds of *Ricinus communis* were crushed together and bandaged to treat small skin swelling with pus resulting due to poisonous spines. The bark and leaf of *Cadaba rotundifolia* and *Withania somnifera* were concocted together to treat extended flow of menstruation. Fruit and leaf of *Cucumis dipsaceus* and *Commicarpus sinuatus* were concocted together and taken orally to treat gonorrhoea. An oily extract from the leaf of *Kleinia longiflora* was boiled and mixed with crushed fresh leaf of *Cadaba rotundifolia*. It is used to massage paralyzed body part every morning and evening to improve nerve function. It was also reported that the fresh leaf of *Gloriosa superba* was crushed with succulent stem of *Caralluma speciosa* and applied on the starting point of gangrene so as to prevent its’ spreading to the rest of the body.

The fresh leaves of three plants i.e. *Steganotaenia araliacea*, *Grewia bicolor*, and *Cissampelos mucronata* were concocted together and taken orally for a person having mental disturbance and body burning feeling. This was the only remedy made from mixture of three plant species. According to traditional healers’ report this remedy has body cooling effect, giving good sleep and mental stability when a small cup of solution is taken in the evening. Some of the traditional healers mentioned that the use of multiple therapies in traditional remedial preparation could increase the efficacy of traditional medicine for the corresponding health problem. According to [50], the use of more than one plant species to prepare a remedy for ailments is attributed to additives or synergistic effects during ailment treatment.

In addition, different plant parts from a single species were prepared in similar ways to treat different types of aliments. For example, the infusion from fresh leaf and seed of *Azadirachta indica* is taken orally to treat malaria and intestinal parasites; the leaf and root of *Craterostigma plantagineum* were concocted together and taken orally to treat liver disease and diarrhoea; leaf and jel of *Aloe harlana* were concocted together and used as an ointment to treat skin and hair fungi. Some remedial preparations need admixture like oil, honey and sugar. These could increase the adhesive nature of remedies particularly for dermal ailments. They also reduce some side effects like bitterness, vomiting; and improve the taste of remedies. Other studies [5,11,23,25,27] also reported about the use of admixtures in remedial preparations for same effects.

Both internal (55.1%) and external (44.9%) routes were used for application of the medicines. For internal application, the most common route was oral that accounted for 45.7% and that of external was dermal ointment which accounted for 12.1% (Table 6). Though, more diversified usage was reported for external use (nine different ways), oral route of administration accounts the highest percentage. Some more studies reported that oral route of administration is the most common [15,25-27,34].

**Table 6:** Routes of administration

**Internal**	**No. of preparations**	**Percentage**	**External**	**No. of preparations**	**Percentage**
Oral drink	64	45.7	Dermal ointment	17	12.1
Oral eaten	6	4.3	Dermal bandage	16	11.4
Eye/internal	4	2.9	Buccal cavity	11	7.9
Ear/internal	2	1.4	Herbal bath	9	6.4
Vaginal/Internal	1	0.7	Drenched	4	2.9
Total	**77**	**55.10%**	Dermal rubbing	2	1.4
			Smoke bath	2	1.4
			Steam bath	1	0.7
			Massage	1	0.7
			**Total**	**63**	**44.90%**

There was no consensus on the dosage used and frequency of medication among the traditional healers. Most of them reported that the dose given to patients depended on age, physical and health conditions. For example, a small piece of an aphrodisiac fresh rhizome of *Kleinia abyssinica* can be enough if properly chewed and swallowed few hours before sexual performance to increase sexual performance of physically weak and less weighted person/man whereas considerably large sized rhizome is needed for same effect for stronger and heavy weighted person/man having problem of sexual dysfunction. Lack of precision and standardization was mentioned as a global drawback of traditional healthcare system [4,27,45]. Similarly, in this study where internal route of application accounts 55.1%, lack of precision can be taken as the major drawback.

Overdose of remedies was also reported to bring adverse effects like, diarrhoea, vomiting, abdominal pain, unconsciousness, and fainting of the patient. During such incident, the traditional healers use different antidotes for reversing adverse effects like, “hoja” a local hot beverage prepared from milk and pericarp of coffee berry, coffee, honey and milk. The same pattern of using antidotes was reported for other cultural groups elsewhere in Ethiopia [5,9,11,26,45].

## Conclusion

Overall, this comprehensive ethnomedicinal study showed that the community in Harla and Dengego Valleys rely on considerable number of traditional medicinal plant species to treat wide spectrum of human ailments and are knowledgeable about the identities and applications of medicinal plants. The majority of medicinal plant species were harvested for their leaves so poses no significant threat to the natural vegetation of the study area. Out of the total ethnomedicinal plant species identified in this study some were endemic plant species like, *Aloe harlana*, *Aloe mcloughlinii*, *Gomphocarpus purpurascens*, *Indigofera ellenbeckii* and *Rhynchosia erlangeri*. Among these endemic medicinal plant species, *Aloe harlana* was found only in this study area. It had been named after the prehistoric Harla. It was the first time for the local community to get this information. They were highly excited and encouraged to further conserve and safeguard such valuable medicinal plant species within their wide scale conservation activities.

Plant species like, *Aloe megalacantha, Cissampelos mucronata, Ocimum lamiifolium, Tamarindus indica, Lawsonia inermis* and *Withania somnifera* scored a high frequency of citations (>50%) among the medicinal plant species which were marketable in the open market places. Such benefits of plants in the primary healthcare system, income generation and higher frequency of citation could be considered as a good opportunity for the support of their livelihood. Therefore, the out put of this comprehensive ethnomedicinal knowledge will encourage the community to conserve, manage and sustainable use the medicinal plant species.

The binomial test on the depth of ethnomedicinal knowledge between younger and elderly informants showed a significant difference. Many young people were less knowledgeable about the variety and value of traditional medicinal plants. This showed the level of deterioration of ethnomedicinal knowledge in this prehistoric study area. It is, therefore, necessary to preserve this indigenous knowledge on traditional medicines by proper documentation, identification of plant species, herbal preparation and dosage. In addition, it should be followed with phytochemical and pharmacological analyses in order to give scientific ground to the ethnomedicinal knowledge.

## Competing interests

We declare that we do not have competing interests.

## Authors’ contributions

AB carried out the field study, identified the medicinal plant species, analyzed the data and wrote the manuscript. NB carried out the field study with AB, revised the manuscript, and provided considerable input. Both of us read the final manuscript and agreed on its submission.
